# 25th anniversary of the Department of Cardiac Surgery at the University Medical Center Maribor: advancing hearts, changing lives: A quarter century of commitment

**DOI:** 10.1007/s00508-024-02323-7

**Published:** 2024-01-29

**Authors:** Miha Antonic, Anze Djordjevic

**Affiliations:** Department of Cardiac Surgery, University Medical Centre Maribor, Ljubljanska ulica 5, 2000 Maribor, Slovenia

**Keywords:** History of medicine, Cardiac surgery, Education, Research, Development

## Abstract

In 1996, Slovenia witnessed a profound transformation in its cardiac care landscape with the establishment of the Department of Cardiac Surgery at the University Medical Centre Maribor. This momentous milestone heralded the birth of the nation’s second heart surgery center revolutionizing cardiovascular care accessibility. Today, the Department of Cardiac Surgery stands as a regional hub, delivering specialized cardiac surgical services to Slovenia’s northeastern region and beyond. Its unwavering commitment to excellence, patient-centered care, and adherence to international guidelines reflects its dedication to providing top-tier cardiac care. As the department commemorates its 25th anniversary, this article offers a reflective overview of its establishment, development, growth and future trajectory for further development in an ever-changing era of cardiovascular medicine. The article also highlights the department’s active involvement in international collaborations, scientific research, medical education, and innovations in minimally invasive cardiac surgery.

## Introduction

In 1996, the landscape of cardiac care in Slovenia changed with the establishment of the Department of Cardiac Surgery at the University Medical Centre Maribor. This marked the birth of the nation’s second heart surgery center, a monumental step in advancing cardiovascular care accessibility. As this department commemorates its 25th anniversary, it is not just a celebration of time passed but a reflection on the profound impact it has had on cardiac care and the journey that led to its inception. The foundation of the department was not a simple feat; it was a convergence of determination, collaboration, and a shared vision among various medical professionals and institutions. Our journey was punctuated by remarkable milestones—from the first open-heart surgery conducted in 1996 to the pioneering procedures, research contributions, and educational initiatives that followed.

As far back as 1954, the then head of the Department of Thoracic Surgery, Janko Držečnik, performed the first operation for constrictive pericarditis and a digital commissurotomy of a stenosed mitral valve in Maribor. Almost concurrently with Professor Košak in Ljubljana, he collaborated with experts from the Electromechanical Club of the Maribor Car Factory to develop Maribor’s version of the extracorporeal circulation apparatus, named “Pulmocor” [1, 2]; however, the circumstances in Slovenia and Yugoslavia at that time were not conducive to the development of cardiac surgery in Maribor. The first open-heart surgery in Maribor had to wait for nearly 40 more years, until 1996.

## The beginning: building the foundation

In the mid-1990s, the conditions within the hospital, which had already embraced several pivotal diagnostic cardiological methods, such as transesophageal echocardiography and coronary angiography, not only laid the groundwork but also provided a compelling mandate for the initiation of cardiac surgical endeavors.

The exceptional relations between Maribor’s cardiac surgeons and cardiologists provided the bedrock of collaboration. This camaraderie was not confined to professional interactions; it was nurtured by a common purpose to enhance patient outcomes and push the boundaries of cardiovascular medicine. The willingness of the University Medical Center Ljubljana to participate in the establishment of a new center in Maribor showcased the unity and shared mission within the medical community. This spirit of collaboration underscored the interconnectedness of medical institutions and their collective commitment to advancing healthcare delivery.

Last but certainly not least, the courage of the hospital management was the vital catalyst that propelled this vision from concept to reality. Their steadfast belief in the potential of cardiac surgery and their willingness to overcome challenges made them the true architects of this transformative endeavor.

In the mid-1990s, as these factors converged, the stage was set for the setting up the Department of Cardiac Surgery at the University Medical Center Maribor. By 1996, the final preparations, purchase of equipment, training of the team and agreements on the scope of work and the program of heart operations were underway.

As a result, the first open-heart operation with cardiopulmonary bypass was performed in Maribor on 7 June 1996. The procedure was carried out by a team of cardiac surgeons from Ljubljana with the assistance of cardiac surgeons from Maribor. This marked a pivotal moment in the development of the second cardiac surgery center in Slovenia.

## Pioneering steps: the early development and subsequent independence

In January 1997, Maribor initiated a regular cardiac surgery program, with patients initially placed in the surgical intensive care unit until their condition stabilized. Subsequently, they were transferred to the Department for Cardiology, where a team of internists, cardiologists, and cardiac surgeons oversaw their care. By mid-1997, a milestone of 50 successful operations was achieved. During the latter half of that year, the hospital formally established the Division of Cardiac Surgery within the surgical service. This division utilized rooms on the third floor of the surgical tower, previously repurposed from the Department of Pediatric Surgery. Simultaneously, a specialized nursing team commenced training.

Operations, initially conducted under the guidance of Ljubljana’s cardiac surgery team, were initially scheduled in the afternoon and nighttime hours due to the absence of their own operating room, sharing the operating room with other branches of surgery, especially thoracic surgery; however, starting from mid-1998, an operating room was dedicated to cardiac procedures and hence morning procedures were also introduced.

On 1 June 1999, the Division of Cardiac Surgery underwent a name change to become the Department of Cardiac Surgery. Substantial renovations were undertaken to adapt the vacated Department of Pediatric Surgery premises to the specific requirements of cardiac surgery. This transformation enabled the growth of a comprehensive operative team that, aided by Ljubljana’s cardiac surgeons, progressively performed an increasing number of operations. Their responsibilities expanded to include patient preparation before procedures and postoperative care. By the close of 2000, Maribor achieved full autonomy in its cardiac surgery endeavors.

The volume of open-heart surgeries displayed a consistent upward trajectory: 4 surgeries in 1996, 95 in 1997, 111 in 1998 and 134 in 1999. Moreover, the complexity of cases also rose, encompassing patients with intricate pathologies.

Starting in 2000, a notable milestone was the surgical team’s attainment of autonomy, no longer requiring the help and guidance from Ljubljana. Over the span of 4 years, UMC Maribor has effectively created an independent and prosperous cardiac surgery program.

Major surgical milestones were: first apicoaortic conduit (2008), first mini-sternotomy aortic valve replacement (2010), first mini-thoracotomy aortic valve replacement (2014), first aortic valve repair (2017), first minimally invasive direct coronary artery bypass (2019), first right thoracotomy mitral valve repair (2021) and first total coronary revascularization via left anterior thoracotomy (2023).

## Current position as a regional center

Today the Department of Cardiac Surgery at the University Medical Center Maribor provides specialized cardiac surgical services for the entire northeastern region of Slovenia, covering an area of approximately 800,000 inhabitants. In addition to treating patients from the University Medical Center Maribor, we also serve as the primary referral center for general hospitals in Celje, Slovenj Gradec, Ptuj and Murska Sobota, and occasionally accept patients from other Slovenian hospitals. We address all adult cardiac and major vascular pathologies (with exceptions such as long-term mechanical support for heart failure and transplantation activities). Our current surgical portfolio includes surgery for ischemic heart diseases (with or without cardiopulmonary bypass, multiarterial grafting, minimally invasive myocardial revascularization [3, 4] and surgical treatment of complications, such as ventricular septal defects, myocardial ruptures and left ventricular aneurysms), aortic valve replacement with mechanical or stented bioprostheses, aortic valve repair and valve-sparing aortic root replacement (both reimplantation and remodelling with external annuloplasty) [5], minimally invasive aortic [6] and mitral valve surgery [7, 8], mitral valve repair and replacement [9], surgical treatment of any heart valve endocarditis, surgery for tricuspid or pulmonary valve disease, aortic surgery (aortic dissection, ascending and arch aneurysms including the frozen elephant trunk procedure [10], trauma to the great vessels), surgery for atrial fibrillation [11], pericardiectomy, surgery for cardiac neoplasms, temporary mechanical circulatory support (intra-aortic balloon pump, extracorporeal membrane oxygenation) [12, 13] and carotid endarterectomy. In addition, we provide surgical assistance and standby for various interventional cardiological (percutaneous coronary interventions, transcatheter aortic valve implantations [14, 15], interventional treatment for atrial and ventricular arrhythmias) or radiological (thoracic endovascular aortic repair) procedures. Surgical implantation of pacemakers and automatic defibrillators is also performed at our institution [16] as well as treatment of postoperative deep sternal wound infections [17, 18]. Our focus and ongoing commitment are directed towards achieving excellence in expertise, patient-centered care and safety, following the current Slovenian and international guidelines. Regular scrutiny within the American Accreditation Commission International (AACI) accreditation system (international accreditation standards for healthcare organizations) assesses the quality and safety of our work.

Since its establishment, the department has been dedicated to achieving excellence in professionalism. We have introduced numerous innovations in our field of expertise and are particularly active in the field of minimally invasive cardiac surgery.

The department is located on the 4th floor of Building 1 at the University Medical Center Maribor (Surgery Clinic). It underwent a complete renovation in 2022. It comprises a total of 19 beds in 7 patient rooms, 2 rooms (total of 8 beds) are organized as an intermediate (step-down) intensive care unit, while the remaining rooms are standard ward patient rooms.

The department has two modernly equipped cardiac surgery operating rooms located within the Surgical Block of the Surgery Clinic. Unfortunately, there is no hybrid operating room available momentarily, three extracorporeal circulation membrane oxygenation (ECMO) devices are on site, one of which serves as a backup. Also two ECMO devices are available for both veno-arterial and veno-venous configurations. Additionally, two intra-aortic balloon pumps are available. A transit-time flow measurement device is standardly used in all revascularization procedures.

The Cardiac Surgery Department holds 6 highest-level beds (level 3 intensive care unit) in the newly established Unit for Cardiac Surgery Perioperative Intensive Care Medicine, currently operating within the shared Intensive Care Unit for Surgical Specialties. A complete renovation of the 3rd floor of the hospital section of the Surgery Clinic is planned for 2023–2024, intended to establish a new 10-bed cardiac surgery intensive care unit, with funding already secured from the Ministry of Health of the Republic of Slovenia.

## International engagement

Shortly after its establishment, the Cardiac Surgery Department became actively involved in the international arena through formal and informal collaborations with foreign cardiac surgery centers. These collaborations encompass training exchanges for staff, cooperation in scientific research, professional collaborations, and educational initiatives. Notably, collaborations with the Texas Heart Institute in Houston have facilitated training for several of our surgeons under Professor Gregorič. The Jessa Hospital in Hasselt, Belgium, hosted extended training periods for our surgeons in the field of minimally invasive heart surgery. Collaboration with the University Medical Center in Leiden, the Netherlands, involves cardiovascular multimodal imaging diagnostics. Our surgeons have also engaged in short-term training programs across various European and North American centers.

## Education

Within the Cardiac Surgery Department, rotation for surgical and cardiology residents are conducted. Active participation in the undergraduate and postgraduate education processes of the University of Maribor’s Medical Faculty is ensured by the department staff as lecturers, leaders of seminars and workshops, and examiners. Clinical training sessions in surgery for medical students are carried out. Additionally, numerous foreign students participating in the Erasmus program are hosted by our department. The implementation of postgraduate studies in biomedical technology at the UM Medical Faculty involves the department head, who is also an associate professor and chief mentor.

Scientific research projects see the medical staff actively engaged, assuming leadership or researcher roles in both international and internal research endeavors. Collaborations with various domestic institutions, including the Medical Faculty, Faculty of Chemistry and Chemical Engineering, and Faculty of Mechanical Engineering at the University of Maribor, as well as the Clinical Department for Heart and Vascular Surgery at University Medical Center Ljubljana, are established, alongside international activities. The outcomes of these international and domestic collaborations are manifested through numerous joint publications in impact factor journals. An active role is displayed in the form of editorial board members and reviewers for more than 20 domestic and international professional journals.

## Conclusion

Slovenia undeniably stands to benefit from the second cardiac center, and the collaborative approach adopted by Maribor’s cardiac surgeons with the nation’s leading hospital institutions is of paramount importance. The future progression of Maribor’s cardiac surgery endeavors will emulate globally acclaimed cardiac centers, focusing primarily on synergizing efforts with the Department for Cardiology and Angiology to provide comprehensive care to patients with cardiovascular conditions. The aim of this integration is to provide access to a spectrum of contemporary forms of treatment, spanning from pharmaceutical interventions, percutaneous procedures to intricate open-heart surgeries.
